# Sense of Happiness and Wellness Among Adolescents and Their School Environment

**DOI:** 10.3390/children12010068

**Published:** 2025-01-07

**Authors:** Sigita Lesinskienė, Rokas Šambaras, Ieva Ridzvanavičiūtė, Izabelė Jūraitytė, Severija Skabeikaitė, Urtė Stanelytė, Margarita Kubilevičiūtė

**Affiliations:** 1 Clinic of Psychiatry, Institute of Clinical Medicine, Faculty of Medicine, Vilnius University, 01513 Vilnius, Lithuania; rokas.sambaras@mf.vu.lt; 2 Faculty of Medicine, Vilnius University, 01513 Vilnius, Lithuania; ieva.ridzvanaviciute@mf.stud.vu.lt (I.R.); izabele.juraityte@mf.stud.vu.lt (I.J.); severija.skabeikaite@mf.stud.vu.lt (S.S.); urte.stanelyte@mf.stud.vu.lt (U.S.); margarita.kubileviciute@mf.stud.vu.lt (M.K.)

**Keywords:** adolescents, mental health, wellness, school environment, happiness, bullying

## Abstract

Background: Happiness and health are crucial elements of adolescents’ lives that significantly impact mental well-being and societal engagement. This article hypothesizes that a suitable school environment may be one of the components that can impact students’ subjective feelings of happiness and health. This research aimed to determine the association between a negative school environment, such as experiencing bullying and feeling insecure at school, and students’ happiness and health. Methods: The study was conducted in 2023, surveying students in grades 7–10 from two Lithuanian cities and their districts. The study included 1992 students (females 50.2%) with a mean age of 14.53 ± 1.12. Results: It was observed that male students felt healthier and happier than females. Also, male students felt safer at school more often than females. A positive correlation was found between a student’s feeling of safety at school and their happiness and health. Regression analysis revealed that a feeling of safety at school was the most significant positive factor associated with male students’ sense of happiness and health. Also, the most important factors for females were a feeling of safety at school, bullying, and how often teachers stop bullying. Conclusions: It was found that feelings of insecurity at school, experiences of bullying, and how often teachers stop bullying can be associated with students’ subjective feelings of happiness and well-being. School environmental factors can affect students’ happiness and well-being differently depending on gender. It is essential to focus on vulnerable student populations when creating preventive programs to enhance adolescents’ sense of safety in schools.

## 1. Introduction

### 1.1. Adolescence

According to the World Health Organization (WHO), adolescents are defined as individuals within the age range of 10 to 19 years. This demographic constitutes approximately one-sixth of the global population [1]. This phase of life is notably distinctive and marked by substantial physical and emotional development challenges. It encompasses essential changes critical to an individual’s growth and maturation. Several distinctive factors characterize the changes from childhood to adulthood. It functions as a pivotal period influenced by heightened peer interactions, shifts in self-esteem and body image, an increase in experimental behaviors, and additional aspects that contribute to individual development [2,3]. One of the defining features of the adolescent years is the variety of ever-changing emotions that a person, taking steps toward adult life, has to deal with.

### 1.2. Concept of Happiness

Happiness is characterized by the enduring joy individuals experience when they find fulfillment in their lives. It serves as the primary objective pursued by all human beings [4]. Philosophers before our time agreed that happiness was the final aim of a person‘s life, with the following quote attributed to Aristotle: “Happiness is the meaning and purpose of life, the whole aim and end of human existence”. The concept of happiness has long been debated, with many researchers offering varying definitions. Happiness can encompass ideas such as experiencing positive emotions, lacking negative emotions, and achieving overall life satisfaction, as well as including additional dimensions. However, the exact nature of happiness and the means to attain it remain questions that have yet to be definitively answered [5,6]. The concept of happiness for children and adolescents is inherently broad and subjective. A study exploring this idea in children aged 5 to 12 uncovered various definitions of happiness. Commonly, happiness is defined in terms of “positive feelings”, “freedom from violence”, “socializing with friends”, and “having free time” [7]. Another study involving middle adolescents found that as many as 22 different definitions of happiness and contentment. The most frequently cited definitions encompassed concepts such as “emotions”, “satisfaction”, “relationships”, and “harmony” [8]. However, the assessment of adolescent happiness presents significant challenges. A systematic review conducted in 2022 indicated that one of the considerable challenges in analyzing adolescent happiness lies in its measurement. A variety of instruments are utilized to evaluate this construct, prompting the question as to whether a definitive “gold standard” exists for the measurement of adolescent happiness. However, a single question about happiness may be sufficient for the assessment, indicating the adolescent’s internal state [9].

### 1.3. Factors That Impact the Happiness of Adolescents

The scientific literature indicates that a variety of factors influence the happiness of adolescents. The research evidence emphasizes the critical importance of friendly relationships as a primary contributor to individual happiness [10]. A study conducted in the United Kingdom in 2007 identified a direct correlation between the quality of interpersonal relationships and the levels of happiness experienced by individuals [11]. In one study, researchers evaluated adolescents’ reports on different types of social relationships and their overall life happiness. The research findings indicated that the quality of friendships—both positive and negative—as well as self-reported experiences of victimization, uniquely contributed to the happiness levels of adolescents [12]. Another essential factor that significantly impacts adolescents’ happiness is the presence of a safe and suitable environment in which they reside or engage in activities. Research indicates that children or adolescents raised in environments free from emotional or physical abuse experienced greater happiness [13]. Also, research shows that family support is a key predictor of adolescents’ happiness [14,15]. A study in Lithuania in 2010 found that subjective happiness among high school students was higher in students living in the countryside with a large family [16]. Moreover, the availability of free time and the degree of autonomy significantly contribute to the happiness of adolescents. Research has demonstrated that several leisure-related factors, such as the type of activity, duration, cost, and location, influence the overall well-being of this age group [17]. It is important to recognize that the understanding of subjective happiness may vary between genders and the ages of adolescents, and the factors that impact adolescents’ conceptions of happiness may also differ. Several studies suggest that feelings of happiness are similarly varied across gender groups, with a slight advantage observed among children and adolescent males [18]. Another study investigated happiness levels among Canadian adolescents aged 12 to 17. The authors revealed that happiness levels differed between genders, with males reporting a higher degree of happiness than females [19].

### 1.4. The Importance of Happiness for Adolescents

Happiness is an essential attribute for young individuals, as it significantly influences their mental health. Furthermore, it is shaped by the integrity of their cognitive abilities [20]. In recent decades, there has been a notable challenge concerning the psychoemotional and physical health of children and adolescents [21,22]. Research evidence demonstrates that there are significant correlations between happiness and various health outcomes. Specifically, individuals who experience higher happiness levels are more likely to participate in routine physical exercise, maintain consistent and quality sleep habits, follow a nutritious diet, and refrain from psychoactive substance use or alcohol consumption [23,24]. Research suggests a strong link between happiness and health, indicating that reduced happiness may not only result from illness but could also be a potential risk factor for health issues [25]. Therefore, strategies to enhance adolescent happiness should be considered a crucial public health priority, given the significant implications for the population’s existing and prospective physical and mental health.

### 1.5. Sense of Health

In this article, we examine not only students’ perceptions of happiness but also of health. Adolescent health is a complex idea that is broadly recognized as including much more than just the absence of illness. It represents a comprehensive condition including physical well-being, mental well-being, and wellness [26]. Therefore, this article will use the term “health” as a synonym for a sense of wellness. Certain authors have posited that health and wellness may be regarded as synonymous terms [27]. However, it is essential to understand that wellness is not merely static. While it encompasses physical, psychological, and social well-being, wellness also fundamentally involves active engagement in specific constructive activities, habits, and lifestyles [28].

### 1.6. The Association Between the School Environment and the Happiness and Health of Students

It is widely acknowledged that adolescents worldwide spend considerable time within educational institutions, which are often regarded as second homes. Considering this, we hypothesized that the school environment could significantly impact students’ perceptions of happiness and health. The school environment is understood to encompass not only the educational process but also interactions with peers and teachers. It serves as a space where students can experience safety, understanding, and a sense of being heard. Therefore, we consider that factors contributing to students’ subjective happiness and well-being may be a safe, educational environment, including the absence of bullying, the establishment of a secure atmosphere, and the proactive engagement of both students and educators in initiatives to prevent bullying. Consequently, it is understandable that the environment in which young individuals spend considerable time significantly influences their identity and emotional state. Moreover, teenagers are particularly receptive to external triggers, rendering them especially susceptible to the diverse atmosphere of a school setting. Studies have demonstrated significant correlations between safe school environments and students’ overall happiness [12,29]. A study conducted in Greece revealed that positive peer relationships, the prevention of bullying, and concern for diversity are significantly linked with the subjective well-being of students [30]. Also, research indicates that a positive school environment is associated with a reduction in bullying, an improvement in academic performance, and the development of stronger interpersonal relationships among students [31]. Moreover, higher education is linked with happiness, so it is important to motivate students to study more, and a suitable school environment could help achieve a better education [4]. Adolescence is a time when the influence of peers significantly emerges as a primary motivating factor in individuals’ lives. Consequently, establishing reliable and trusting friendships becomes crucial in fostering a healthy and positive mindset [32]. It is also crucial to mention that the COVID-19 pandemic has profoundly impacted the school environment. When it began in 2020, most countries closed their schools, prompting students to switch to remote learning. This shift created significant challenges in organizing the learning process. Digital distance learning became a crucial method for controlling the spread of infections while ensuring educational continuity. Although online education and virtual communication have important benefits, the increased frequency and duration of internet use during quarantine periods may have negatively affected students’ mental and emotional well-being [33]. Following the quarantine’s conclusion, most students returned to classes. This transition represented a novel experience for most, necessitating a period of adjustment [34].

### 1.7. Aim of the Study

There remains a noticeable gap in the research regarding the relationship between school environments and students’ overall sense of wellness and happiness. Our research investigates the subjective perceptions of happiness and health among middle adolescents in Lithuania, with a particular emphasis on the influence of the school environment on these perceptions. The aim is to expand our understanding of the possible connections between positive school environments—such as the absence of bullying, a sense of security, and teachers’ involvement in bullying prevention—and happiness and health. This research is particularly valuable in the Baltic States, where such studies are significantly lacking.

Our study aimed to determine the association between a negative school environment, such as experiencing bullying and feeling insecure at school, and Lithuanian students’ subjective feelings of happiness and health. As researchers, we hypothesized that (1) students’ sense of wellness and happiness would differ between genders and between younger and older students [18]; (2) bullying experiences and feelings of safety at school would vary between genders and between older and younger students [35]; (3) feelings of insecurity at school and bullying experiences would negatively impact students’ feelings of wellness and happiness [12,29]; and (4) the effect of school environmental factors on students’ happiness and well-being will differ between students’ genders [36].

## 2. Materials and Methods

### 2.1. Procedure and Participants

The research was carried out in two cities in Lithuania—Vilnius, Klaipėda, and their associated districts. The research included three educational institutions in each city, comprising two gymnasiums (grades 9 to 12) and one pro-gymnasium (grades 1 to 8). Furthermore, one gymnasium was surveyed in each of the city districts. To achieve the study’s objectives, interviews were conducted with students in grades 7 through 10, corresponding to the ages of 13 to 17.

For this study, school selection was carried out considering urban population density and geographic location criteria. Vilnius, the capital city, is the largest urban center in Lithuania, while Klaipėda ranks as the third largest city in the nation. In September and October 2023, eight chosen school administrations were asked to take part in a confidential survey and agreed to participate in the study. In response to the ongoing investigation, the school administration communicated with the parents of the students and obtained their official consent. In the autumn of 2023, the researchers conducted visits to schools to interview students. Adolescents whose parents granted approval were administered questionnaires and were supplied with detailed information regarding the objectives of the study. They were informed that their participation was voluntary and that they had the freedom to withdraw from the study at any time. The initial page of the questionnaire contained a section on informed consent for the participants. The questionnaire proceeded only after the participants formally indicated their willingness to partake in the survey. The questionnaires were filled out by the students in a classroom setting, an activity that took approximately the duration of one class period. The information provided explicitly outlined the objectives of the study and emphasized our commitment to maintaining ethical practices, which include guaranteeing the anonymity and confidentiality of all participants.

### 2.2. Measures

A questionnaire designed by the authors, comprising four distinct sections, was employed to carry out the research.

#### 2.2.1. Demographic Data

In the initial part of the survey, we gathered demographic information from the participants by asking the following questions: (1) In which city do you live? (the options were Vilnius, Klaipėda, Vilnius district, and Klaipėda district); (2) In which grade are you studying? (the options were 7, 8, 9, or 10); (3) What is your age?; (4) What gender are you? (the options were male, female, or other gender). It is important to note that responses categorized as ‘other’ were excluded from the statistical analysis of gender, as they constituted less than 0.5% of the sample size).

#### 2.2.2. Well-Being of Students

In the second section of the questionnaire, we posed two questions regarding students’ overall well-being: (1) Do you consider yourself to be healthy? (response options: yes, moderate, or no); (2) Do you consider yourself to be happy? (response options: yes, moderate, or no).

#### 2.2.3. Bullying at School

In the third section of the questionnaire, we asked students several questions regarding their experiences with bullying at school over the previous six months. (1) How often have you been bullied at school? (response options: not at all, less than once a week, more than once a week, or almost every day); (2) How often have you bullied others at school? (response options: not at all, less than once a week, more than once a week, or almost every day).

#### 2.2.4. Sense of Safety at School

In the final section of the questionnaire, students were asked three questions regarding their sense of safety at school: (1) I feel safe at school (response options: not at all, sometimes, often, always); (2) Teachers or other adults at school make an effort to stop bullying (response options: not at all, sometimes, often, always); (3) Other students take action to stop bullying (response options: not at all, sometimes, often, always).

### 2.3. Data Analyses

All statistical analyses for this study were conducted utilizing SPSS version 29. Continuous variables were assessed for normal distribution using the Kolmogorov–Smirnov or Shapiro–Wilk tests. The findings of the study are reported as the mean and the standard deviation (± or SD). The variations among the analyzed groups were assessed using Student’s *t*-test or the alternative non-parametric method, the Mann–Whitney U test. Pearson’s chi-squared test (χ^2^) was utilized to evaluate whether a statistically significant difference exists between the observed frequencies and the expected frequencies in one or more categorical variables. Spearman’s rank correlation test assessed the relationships between numerical and ranked variables. A linear regression model was employed to assess the association between a dependent variable and one or more independent variables, while also considering possible confounding factors. Several key criteria must be considered when determining the suitability of a linear regression model for deriving valid conclusions. These indicators include a coefficient of determination (R^2^) greater than 0.20, a Cook’s distance of less than 1, and an ANOVA *p*-value of less than 0.05. The alpha level for statistical significance was established at *p* < 0.05.

## 3. Results

### 3.1. Demographics Data of the Sample

The research encompassed eight educational institutions, achieving response rates of 78% and 95%, respectively, across these schools. The sample comprised 1922 students, with a mean age of 14.53 years (standard deviation ±1.12), ranging from a minimum age of 12 to a maximum of 17 years. The distribution of respondents by gender was nearly equal, with 929 females (50.2%) and 921 males (49.8%). The average ages were 14.55 ± 1.13 for females and 14.50 ± 1.12 for males (*p* = 0.921). There was a similar distribution of students between two areas: Vilnius city and Vilnius district, which accounted for 1035 students (53.9%), and Klaipėda city and Klaipėda district, with 887 students (46.1%). The number of students in each grade varied, ranging from 406 students (21.7%) to 508 students (27.1%). Table 1 contains more detailed demographic information.

### 3.2. Student Wellness and Sense of Happiness

After analyzing the survey data, it was found that when asked, “Do you consider yourself to be healthy?” more than half of the students (1127 (62.9%)) reported feeling healthy. Meanwhile, 528 students (29.5%) considered themselves moderately healthy, and 136 students (7.6%) felt unhealthy. When the students were asked about their happiness, only half (806 (48.1%)) indicated they felt happy. A third of the respondents (604 (36.1%)) reported feeling moderately happy, while 264 students (15.8%) stated they were unhappy.

When comparing the perception of health between genders, we used Pearson’s chi-squared test (χ^2^). It was noted that females were significantly less likely to indicate they felt healthy (455 (52.5%)) compared to males, of whom 634 (73.6%) reported feeling healthy, *p* < 0.001. Similar results were observed when asking students whether they considered themselves happy: 494 (59.2%) males indicated that they felt happy, while only 285 (36.5%) females stated that they felt happy, *p* < 0.001.

When comparing the perception of health between two groups of students, we also used Pearson’s chi-squared test (χ^2^). The data indicated that there was no statistically significant difference observed between students in a progymnasium (grades 7–8) and those in a gymnasium (grades 9–10). A total of 451 students from the progymnasium (62.0%) reported considering themselves healthy, while 602 gymnasium students (63.0%) felt the same way, *p* = 0.776. Also, the data showed that there was no statistically significant difference in happiness levels between progymnasium and gymnasium students. In particular, 359 students from progymnasiums (49.4%) indicated that they felt happy, while 421 students from gymnasiums (46.5%) reported similar feelings, *p* = 0.383. Table 2 presents comprehensive information regarding the wellness and happiness of students.

### 3.3. Students’ Experiences of Bullying at School

Three-quarters of the students (1448 (76.7%)) indicated that they had not experienced bullying in an educational environment in the past six months. 286 students (15.1%) reported being bullied less than once a week, 95 students (5.0%) experienced bullying more than once a week, and 60 students (3.2%) reported being bullied almost every day. The replies from the students regarding the question “How often have you bullied others at school?” showed a similar distribution. The results indicated that 1430 (76.0%) respondents reported they had never engaged in bullying behavior. Furthermore, 15.5% acknowledged that they had bullied others less than once a week, while 5.1% indicated more than once a week and 3.5% almost every day.

We used Pearson’s chi-squared test (χ^2^) to analyze the frequency of bullying across genders. Both males (699, 77.4%) and females (706, 77.2%) reported that they had not experienced bullying at school in the past six months, *p* = 0.405. However, when comparing how often students themselves bullied others over the past six months by gender, we found statistically significant differences; females (730 (80.1%) never bullied other students, compared to males (649 (72.2%)), *p* < 0.001.

A comparative study of bullying experiences among students from the progymnasiums and gymnasiums over the previous six months found that adolescents in the gymnasiums were significantly more inclined to state that they had not experienced bullying in school. Specifically, 814 gymnasium school students (81.5%) indicated they had not been bullied, while 598 progymnasium school students (70.7%) reported the same, *p* < 0.001. A similar outcome was achieved when comparing how often gymnasium and progymnasium students bullied other students. It was found that gymnasium students were much more likely not to have bullied others at all (775 (77.8%)) compared to progymnasium students (619 (73.5%)), *p* = 0.002. Table 3 provides a more comprehensive overview of students’ experiences with bullying in school.

### 3.4. Students Sense of Safety at School

A third of students (632 (32.9%)) indicated that they often feel safe at school, 516 (27.1%) students—always, 512 (26.9%)—sometimes, and 243 (12.8%)—not at all. Also, almost a third (579 (30.9%)) of the surveyed students indicated that teachers or other adults at school sometimes make an effort to stop bullying, and 464 (24.8%) students indicated not at all, 449 (24.0%)—often, and 382 (20.4%)—always. Half of the students (924 (48.9%)) in the survey noted that not at all other students take action to stop bullying situations, 648 (34.3%) students indicated sometimes, 213 (11.3%) said often, and only 104 (5.5%) indicated always.

Pearson’s chi-squared test (χ^2^) revealed that female students were notably less inclined to always feel safe at school compared to their male counterparts (20.0% vs. 34.3%), *p* < 0.001. Also, male students were statistically significantly less likely to indicate that other students always take action to stop bullying situations than female students (7.1% vs. 3.7%), *p* < 0.001.

Safety at school was statistically assessed, and it was found to be significantly different between gymnasium and progymnasium students. Gymnasium students reported always feeling safe at school more often than progymnasium students, with percentages of 29.7% and 23.5%, respectively (*p* < 0.001). Also, statistically significantly, gymnasium students indicated less often that other students do not take action at all to stop bullying situations than progymnasium students (45.0% vs. 53.9%), *p* < 0.001. More comprehensive comparisons of feelings of safety at school across different genders and student grades are presented in Table 4.

### 3.5. Student Wellness and Sense of Happiness Association with Demographic Data, Experiences of Bullying, and Sense of Safety in School

Spearman’s rank correlation indicated a moderate positive relationship between student wellness and feelings of happiness (for females, ρ_s_ = 0.487, and for males, ρ_s_ = 0.470, *p* < 0.001). Also, a weak positive correlation was observed between student wellness and the perception of safety within schools (for females, ρ_s_ = 0.260, and for males, ρ_s_ = 0.263, *p* < 0.001). A comparable weak positive relationship was observed between students’ feelings of happiness and their sense of safety at school (for females, ρ_s_ = 0.334, and for males, ρ_s_ = 0.279, *p* < 0.001). Furthermore, a weak negative correlation was found between female students’ perceptions of happiness and the bullying they had faced in the previous six months (ρ_s_ = −0.245, *p* < 0.001), while a very weak negative correlation was detected among male students (ρ_s_ = −0.177, *p* < 0.001). Also, research indicated that the students’ feelings of safety in school showed a weak positive correlation with the frequency at which teachers or other adults attempted to prevent bullying (for females, ρ_s_ = 0.343, and for males, ρ_s_ = 0.355, *p* < 0.001). An expanded correlation matrix is presented in Table 5.

A linear regression was calculated to predict student wellness and sense of happiness based on the experience of bullying, bullying of others, safety in school, bullying prevention from the side of teachers and students, age, and gender. The previously presented variables statistically significantly predicted male student sense of wellness (F = 36.353, *p* < 0.001, R^2^ = 0.278, VIF for all factors was ≤5) and female student sense of wellness (F = 33.636, *p* < 0.001, R^2^ = 0.275, VIF for all factors was ≤5). According to the linear regression analysis, sense of happiness (β = 0.456, *p* < 0.001) and safety at school (β standardized coefficient = 0.107, *p* < 0.001) had the most substantial influence on male students’ sense of wellness. Meanwhile, among female students, the most significant regressors were a sense of happiness (β = 0.437, *p* < 0.001) and teachers stopping bullying (β = 0.086, *p* = 0.020). Table 6 presents the full linear regression analysis that predicts students’ sense of wellness using demographic information, experiences with bullying, and feelings of safety at school.

A further linear regression significantly predicted male students’ sense of happiness (F = 40.151, *p* < 0.001, R^2^ = 0.298, VIF ≤ 5) and that of female students (F = 38.656, *p* < 0.001, R^2^ = 0.305, VIF ≤ 5). The linear regression analysis revealed that a sense of wellness (β standardized coefficient = 0.443, *p* < 0.001) and feelings of safety at school (β standardized coefficient = 0.152, *p* < 0.001) significantly influenced male students’ sense of happiness. Meanwhile, among female students, the most significant regressors were a sense of wellness (β = 0.419, *p* < 0.001), feelings of safety at school (β = 0.200, *p* < 0.001), and bullying experiences (β = −0.129, *p* < 0.001). Table 7 shows the complete linear regression predicting students’ sense of happiness based on demographic data, experiences of bullying, and the feeling of safety in the education environment.

## 4. Discussion

Our research indicated that only 48.1% of the Lithuanian schoolchildren surveyed reported feeling happy, whereas slightly over half, at 62.9%, expressed a sense of being healthy. It was noted that female respondents demonstrated a markedly higher tendency to report feelings of unhappiness and poor health compared to their male counterparts. Scientific research has demonstrated a varied distribution of happiness and health across different genders. Numerous studies indicate that female gender may serve as a weak predictor of lower happiness [37,38]. Most research studies concentrate on adult populations, resulting in inconclusive findings regarding adolescents. However, studies examining adolescents’ happiness or life satisfaction levels have indicated that girls were unhappier or less satisfied with their lives than boys [39]. A research study carried out in Norway, which involved adolescents between the ages of 13 and 18, revealed that boys showed greater levels of self-esteem and life satisfaction compared to girls [40]. However, research has demonstrated that there is no statistically significant difference in the levels of happiness or overall well-being among adolescents [41,42]. The findings from the meta-analysis of 46 separate studies, encompassing a total of 11,772 students (ranging from ages 9 to 20), indicated that both male and female adolescents seem to have similar levels of overall life satisfaction [18]. Although gender unequivocally does not exhibit a direct correlation with happiness levels among adolescents, this finding does not preclude the potential for gender to affect how different variables impact the subjective feelings of happiness experienced by boys and girls [43]. These variations may also be linked to particular aspects of life happiness. For example, in one study, adolescent girls were more satisfied with their schoolwork, families, and friends than boys [41]. However, it is important to mention that some studies suggest that, over the last few decades, the societal position of women has experienced considerable transformations in multiple areas. Consequently, the level of happiness perceived by females may also have evolved in response to these social transformations. This is particularly evident among adolescents who are navigating critical transitional phases in their lives [18].

The results of this research suggest that there is no statistically significant relationship between students’ ages and their happiness, as shown through correlation and linear regression analyses. However, research indicates that individuals may experience changes in their happiness, wellness, and satisfaction levels during the shifts from childhood to adolescence and then from adolescence to young adulthood. Many publications suggest that happiness, well-being, or life satisfaction decreases from childhood to adolescence [44,45]. For instance, a research study carried out in Germany in 2007 examined a group of 1274 students between the ages of 11 and 16. The findings revealed that older adolescents reported lower life satisfaction and overall well-being levels than their younger counterparts [39]. The observed decline in happiness during adolescence can be attributed to various developmental changes during this period. These changes encompass brain development, hormonal changes, emotional responses, cognitive development, behavioral adjustments, and transformations in interpersonal relationships [39,46]. It is essential to recognize that age differences may significantly influence the conceptualizations of happiness among children and adolescents. The comprehension of happiness in young children will likely differ markedly from that of adolescents, highlighting the need to consider developmental stages when examining perceptions of joy and well-being [43,47].

Our research indicates that approximately 25% of students reported experiencing bullying within their school environment. Bullying is characterized as a pattern of intentional aggressive behavior, whether perpetrated by an individual or a group, directed toward another individual. This behavior typically reflects a significant imbalance in power, which may arise from differences in physical strength, social status, or other pertinent factors. Bullying encompasses various forms of harmful behavior, including verbal abuse such as name-calling and physical violence. Additionally, it manifests through harassment via telephone calls, text messages, or social media platforms, a phenomenon commonly referred to as cyberbullying. Furthermore, studies demonstrate that the rates of bullying among young people differ significantly from one country to another, with prevalence ranging from 5.1% to 41.4% [47]. Our research indicates that boys exhibit a higher tendency to engage in bullying behaviors compared to girls. Other studies also confirm that boys are more frequently identified as bullies or victims of bullying compared to girls. In contrast, girls tend to be more often recognized as victims of bullying [48]. It is noteworthy that bullying phenomena are more prevalent among male students, who frequently exhibit a greater tolerance for aggressive behavior. On the other hand, indirect types of bullying, like spreading rumors and social isolation, are seen more often among females [47]. Our research indicated that younger students are at a greater risk of experiencing bullying. The results are aligned with current research that investigates the incidence of bullying in young people. A study carried out in Finland highlighted that instances of victimization and bullying occur more frequently in primary schools compared to middle and secondary schools [49].

The results of this study suggest that bullying has a detrimental effect on the happiness and overall well-being of adolescents. Other research findings support the notion that adolescent bullying is prevalent and emphasize that increased happiness among adolescents corresponds with a reduction in experiences of violence and conflict, including incidents of quarreling and bullying [50]. Also, research has increasingly shown that bullying can significantly affect both behavior and health [51]. Numerous scholars have indicated that both bullying and cyberbullying can result in enduring consequences, impacting individuals in both the short term and the long term. These adverse effects can significantly influence overall adolescent development [52,53,54]. Also, bullying has been recognized as a key contributor to the recent surge in depression, anxiety, and suicide rates among young people over the last ten years [51]. Numerous articles indicate that bullying can either lead to or contribute to mental health issues and unhappiness among adolescents. This impact is particularly significant when an adolescent occupies dual roles as a victim and a perpetrator. While there remains ongoing debate regarding whether bullying is the primary cause of the deterioration in mental health among young individuals, research consistently underscores its detrimental effects [55]. Victimization raises the likelihood of depression and suicidal thoughts in victims and can undermine their self-esteem, emotional competence, trust in others, and overall engagement in life [53]. Furthermore, being bullied—both in person and online—correlates with a decline in positive feelings like hopefulness, overall joy, and school-related happiness. It also has a detrimental effect on specific aspects of life satisfaction [50]. However, some authors have found that bullying behaviors relate differently to happiness. Specifically, being a victim is slightly more strongly associated with happiness than being a bully, with face-to-face bullying having a greater impact than cyberbullying [50]. Different organizations, such as the American Academy of Pediatrics (AAP) and the American Academy of Child and Adolescent Psychiatry, highlight the crucial importance of mental health experts and healthcare providers in both preventing and tackling bullying. Offering proactive advice, employing effective screening methods to detect occurrences of bullying, and intervening promptly can greatly improve the well-being of numerous children and their families [51].

A major factor that affected happiness and well-being in our research was the perception of safety in schools. We found a direct correlation between students who felt safe at school and those who reported feeling happier and healthier. It is also important to notice the potential bidirectional relationships between happiness or well-being and school safety. A systematic review encompassing 43 studies revealed that the average prevalence of unsafe school environments is approximately 19%, with reported prevalence rates ranging from 6.1% to 69.1% [56]. Another comprehensive cross-cultural study revealed that 30% of adolescents reported feeling unsafe at school, with notable variations across the countries included in the research. Specifically, among girls, the proportion of those who feel unsafe varies significantly, from 11.5% in Finland to 69.8% in Japan. In the case of boys, the data reflect a comparable pattern, with just 7.7% in Norway reporting feelings of unsafety, while 68.2% in Japan express the same concern [57]. Research indicates that a perception of unsafety within the school environment is correlated with the presence of depressive symptoms among students [58,59]. Also, research indicates that self-harming behaviors are correlated with individuals’ perceptions of safety within the school environment [60]. A study employed the self-reported Strengths and Difficulties Questionnaire to explore the connection between adolescents’ emotional challenges and their sense of safety within the school environment. The results revealed a notable correlation between reported emotional issues and difficulties related to peers and conduct [61]. Research suggests that a heightened sense of insecurity among students in educational institutions may be attributable to the prevalence of bullying or an established culture of bullying within the school environment [62,63]. Another crucial factor that can enhance adolescents’ sense of safety within an educational institution is characterized by the development of good relationships with teachers and the perception of being valued by them [64,65].

The relationship between the well-being of adolescents, their general health, and their feelings of safety and contentment in the school setting is a critical area of exploration in Lithuania and globally. Happiness serves as a significant indicator of robust mental well-being among adolescents. Furthermore, research indicates that individuals who report higher happiness levels often demonstrate improved academic performance and are more likely to succeed in their future endeavors [10]. Not only the feeling of happiness but also wellness can determine whether adolescents feel satisfied. Research has shown a significant link between school dropout rates and Health-Related Quality of Life (HRQOL). Young people who reported one to four sick days and those with five or more days demonstrated lower physical HRQOL than their peers who had not experienced illness [66]. A Lithuanian research paper indicates a correlation between happiness and creativity among young people. It indicates that creativity is linked to well-being, encompassing life contentment, personal development, and positive emotions. Comprehending these relationships can facilitate the development of interventions aimed at fostering creativity and enhancing well-being in educational contexts [67]. Combining a secure learning environment, open communication, and supportive school personnel among adolescents in Lithuanian schools leads to social and subjective well-being. An entirely optimistic perspective on school correlates with greater well-being and positive social interactions among students [68].

Considering these findings, schools in Lithuania should prioritize promoting a culture of well-being that benefits not just individual students but the entire school community. This can involve implementing initiatives designed to improve the school environment, including physical spaces, social interactions, and teaching methods. Additionally, offering professional development opportunities for teachers and staff focused on fostering well-being in the school setting will equip them with the understanding and abilities required to promote a more encouraging and constructive educational atmosphere for every student. Several adjustments must be made at different levels to foster positive school changes that promote adolescents’ well-being and happiness. Interventions carried out in schools or other educational institutions can illustrate how schools can serve as settings that promote the development of students’ well-being and sense of happiness [69]. Also, this could involve reworking the educational system, improving the abilities and understanding of teachers and administrators, and cultivating a sense of community among the school populations. A study conducted in Turkey revealed that victimized pupils who feel a sense of connection to their school environment exhibit fewer emotional difficulties and enhanced well-being compared to their peers who are victimized but do not feel a connection to their school [70]. Some studies indicate that schools should prioritize promoting a sense of belonging at multiple levels within the educational system. This initiative could be implemented through the establishment of school policies, practices, and public communications directed at school communities, such as the articulation of school vision and mission statements [71]. Research demonstrates that the perception of belonging within an educational environment is positively correlated with heightened levels of happiness and enhanced psychological well-being among students [72]. Programs aimed at improving students’ ability to manage their emotions and their skills for co-regulating with classmates and educators effectively promote psychological well-being and social connections among primary and secondary school students [73]. For instance, a study carried out in the United States examined students in the 6th and 7th grades. The intervention emphasized restorative practices and comprised three main elements: 1. Promoting ongoing positive relationships between educators and learners; 2. Enhancing students’ abilities as guided by teachers, concentrating on seven of the eleven fundamental practices; 3. Motivating students to utilize these skills in real situations as they become adept in the seven crucial practices. Following the implementation of these strategies, students reported marked enhancements in school atmosphere, peer connections, and social abilities, and a decline in experiences of cyberbullying victimization [74]. An intervention was conducted in France with the primary objective of improving the school climate and promoting positive coexistence among students. This was achieved by developing effective problem-solving strategies and promoting a culture of absolute intolerance for violence as a fundamental aspect of the school’s identification. The strategies involved institutional collaboration and the commitment of the entire school community. Following the intervention, a significant improvement in the sense of belonging was observed in the intervention group, as indicated by the school climate subscale, compared to the control group [75]. Research demonstrates that teacher–student interactions are critical in enhancing student well-being and satisfaction. High-quality relationships between educators and learners provide a vital support system for sustained educational success. Educator behaviors that foster a sense of support and care for students are categorized as emotional support [76].

## 5. Conclusions

Happiness and wellness are important qualities for young people, and they affect their mental health and are influenced by the integrity of their mental capabilities. Our study found that female students feel less healthy and happy and more often feel unsafe at school than male students. However, no significant difference between older and younger students regarding health and sense of happiness was found. However, bullying was observed to be higher among younger progymnasium students than among older gymnasium students. Research indicates that male students, along with younger students, exhibit a higher propensity for bullying their peers. It was found that younger students felt unsafe at school more often. It was found that feelings of insecurity at school, experiences of bullying, and how often teachers stop bullying can be associated with students’ subjective feelings of happiness and well-being. It is essential to admit that the impact of educational institutions’ environmental aspects on students’ perceptions of happiness and well-being may differ based on gender. Considering these factors, it is crucial to focus attention on more vulnerable student populations when designing preventive programs aimed at encouraging a sense of safety among adolescents in school environments and bullying prevention.

## 6. Limitation

It is essential to recognize that this research has various limitations restricting its ability to examine adolescents’ happiness and well-being thoroughly. The research used a cross-sectional approach, which facilitated the recognition of associations among different factors. Nevertheless, it is significant that the study was not able to establish causal relationships. It is possible that feelings of insecurity and experiences of bullying within the educational environment may contribute to diminished health and well-being among adolescents. Conversely, adolescents who perceive themselves as less healthy or who experience unhappiness are likely to report greater feelings of insecurity at school and may also be at an increased likelihood of facing bullying. One important consideration is that the research employed non-standardized questionnaires to evaluate adolescents’ happiness and health. This issue may result in respondents misunderstanding and inaccurately conceptualizing the definitions of happiness and health. By asking students if they feel happy or healthy, we aim to clarify their current emotions as they experience them during questioning, without framing the responses in terms of abstract concepts of happiness or health. It is crucial to acknowledge that the concepts of “happiness” and “health” function as umbrella terms that encompass broader concepts. Consequently, one cannot make definitive connections solely between these concepts and issues such as bullying or safety within educational environments. Therefore, future research should utilize more objective measurement tools, as the current questions mainly depend on subjective evaluations.

## Figures and Tables

**Table 1:** Student demographic data.

Variable		N	%
Gender			
Male		921	49.8
Female		929	50.2
Cities			
Vilnius city and Vilnius district		1035	53.9
Klaipėda city and Klaipėda district		887	46.1
Classes in which students study			
Progymnasiums	7	454	24.2
	8	406	21.7
Gymnasiums	9	507	27.0
	10	508	27.1

**Table 2:** Comparison of student wellness and sense of happiness between genders and student classes.

Do You Consider Yourself to Be Healthy?								Do You Consider Yourself to Be Happy?						
Variables	Yes		Moderate		No		*p*	Yes		Moderate		No		*p*
	n	%	n	%	n	%		n	%	n	%	n	%	
Gender														
Male	634	73.6	191	22.2	36	4.2	<0.001	494	59.2	244	29.3	96	11.5	<0.001
Female	455	52.5	319	36.8	93	10.7		339	36.5	339	43.5	156	20.0	
Classes in which students study														
Progymnasium classes	491	62.0	236	29.8	65	8.2	0.776	359	49.4	250	34.4	117	16.1	0.383
Gymnasium classes	602	63.0	284	29.7	70	7.3		421	46.5	341	37.7	143	15.8	

**Table 3:** Comparison of students’ bullying experiences at school between genders and student classes.

How Often Have You Been Bullied at School?									
Variables	Not at All		Less than Once a Week		More than Once a Week		Almost Every Day		*p*
	n	%	n	%	n	%	n	%	
Gender									
Male	699	77.4	131	14.5	41	4.5	32	3.5	0.405
Female	706	77.2	144	15.8	43	4.7	21	2.3	
Classes in which students study									
Progymnasium classes	598	70.7	144	17.0	61	7.2	43	5.1	<0.001
Gymnasium classes	814	81.5	138	13.8	32	3.2	15	1.5	
How often have you bullied others at school?									
Gender									
Male	649	72.2	165	18.4	57	6.3	28	3.1	<0.001
Female	730	80.1	118	13.0	33	3.6	30	3.3	
Classes in which students study									
Progymnasium classes	619	73.5	142	16.9	38	4.5	43	5.1	0.002
Gymnasium classes	775	77.8	145	14.6	55	5.5	21	2.1	

**Table 4:** Comparison of sense of safety at school between genders and student classes.

I Feel Safe at School									
Variables	Not at All		Sometimes		Often		Always		*p*
	n	%	n	%	n	%	n	%	
Gender									
Male	104	11.5	176	19.4	315	34.8	311	34.3	<0.001
Female	122	13.2	318	34.3	301	32.5	185	20.0	
Classes in which students study									
Progymnasium classes	139	16.3	243	28.5	271	31.7	201	23.5	<0.001
Gymnasium classes	98	9.8	259	25.8	349	34.8	298	29.7	
Teachers or other adults at school make an effort to stop bullying									
Gender									
Male	222	24.7	269	30.0	198	22.1	208	23.2	0.053
Female	212	23.3	292	32.2	240	26.4	164	18.1	
Classes in which students study									
Progymnasium classes	226	27.0	251	30.0	193	23.0	168	20.0	0.293
Gymnasium classes	229	23.1	309	31.1	245	24.7	209	21.1	
Other students take action to stop bullying situations									
Gender									
Male	459	50.8	276	30.5	105	11.6	64	7.1	<0.001
Female	423	46.2	355	38.8	103	11.3	34	3.7	
Classes in which students study									
Progymnasium classes	453	53.9	271	32.2	74	8.8	43	5.1	<0.001
Gymnasium classes	452	45.0	363	36.2	132	13.1	57	5.7	

**Table 5:** Correlation matrix of student wellness, sense of happiness, demographic data, experiences of bullying, and sense of safety in school.

	Female	Variables							
Male		Health ^a^	Happiness ^b^	Bullying ^c^	Bullying Others ^d^	Safety ^e^	Stopping Bullying (Teachers) ^f^	Stopping Bullying (Students) ^g^	Age
Health ^a^			0.487 **	−0.174 **	−0.081 *	0.260 **	0.196 **	0.151 **	0.019
Happiness ^b^		0.470 **		−0.245 **	−0.107 **	0.334 **	0.159 **	0.096 **	−0.015
Bullying ^c^		−0.197 **	−0.177 **		0.193 **	−0.256 **	−0.156 **	−0.143 **	−0.110 **
Bullying others ^d^		−0.133 **	−0.156 **	0.304 **		−0.107 **	−0.128 **	−0.070 *	0.024
Safety ^e^		0.263 **	0.279 **	−0.183 **	−0.117 **		0.343 **	0.263 **	0.086 **
Stopping bullying (teachers) ^f^		0.136 **	0.157 **	−0.178 **	−0.128 **	0.355 **		0.379 **	−0.044
Sopping bullying (students) ^g^		0.136 **	0.151**	−0.133 **	−0.122 **	0.233 **	0.441 **		0.067 *
Age		−0.039	0.044	−0.123 **	−0.060	0.032	−0.013	0.068 *	

Above the diagonal are Spearman’s correlation coefficients related to female student wellness, happiness, demographic data, bullying experiences, and safety in school. Below the diagonal are Spearman’s correlation coefficients related to male students. * *p* < 0.05; ** *p* < 0.001; ^a^ Do you consider yourself to be healthy?; ^b^ Do you consider yourself to be happy?; ^c^ How often have you been bullied at school?; ^d^ How often have you bullied others at school?; ^e^ I feel safe at school; ^f^ Teachers or other adults at school make an effort to stop bullying; ^g^ Other students take action to stop bullying situations.

**Table 6:** Linear regression predicting students’ wellness from demographic data, experiences of bullying, and feelings of safety in school.

Variable	Do You Consider Yourself to Be Healthy?					
	Male			Female		
	B	SE_b_	β	B	SE_b_	β
Intercept	1.248	0.249		0.609	0.329	
Happiness ^a^	0.357	0.026	0.456 **	0.409	0.036	0.437 **
Bullying ^b^	−0.057	0.026	−0.074	0.028	0.036	0.026
Bullying others ^c^	−0.004	0.025	−0.005	−0.020	0.031	−0.021
Safety ^d^	0.058	0.019	0.107 **	0.053	0.027	0.074 *
Stopping bullying (teachers) ^e^	0.015	0.018	0.030	0.056	0.024	0.086 *
Stopping bullying (students) ^f^	0.003	0.021	0.006	0.053	0.030	0.062
City ^g^	−0.039	0.034	−0.036	0.026	0.044	0.019
Age	−0.022	0.015	−0.046	0.032	0.020	0.053

* *p* < 0.05, ** *p* < 0.001, B = unstandardized regression coefficient, SE_b_ = standardized error of the coefficient, and β = standardized coefficient. ^a^ Do you consider yourself to be happy?; ^b^ How often have you been bullied at school?; ^c^ How often have you bullied others at school?; ^d^ I feel safe at school; ^e^ Teachers or other adults at school make an effort to stop bullying; ^f^ Other students take action to stop bullying situations; ^g^ In which city do you live? Reference values: health = yes, happiness = yes, bullying = almost every day, bullying others = almost every day, safety = always, stopping bullying (teachers) = always, stopping bullying (students) = always, city = Klaipėda city and Klaipėda district, age = 17 years old.

**Table 7:** Linear regression predicts students’ sense of happiness from demographic data, experiences of bullying, and a sense of safety in school.

Variable	Do You Consider Yourself to Be Happy?					
	Male			Female		
	B	SE_b_	β	B	SE_b_	β
Intercept	0.627	0.318		1.387	0.341	
Health ^a^	0.565	0.041	0.443 **	0.448	0.036	0.419 **
Bullying ^b^	−0.025	0.032	−0.025	−0.146	0.038	−0.129 **
Bullying others ^c^	−0.062	0.031	−0.065 *	−0.016	0.033	−0.016
Safety ^d^	0.106	0.024	0.152 **	0.154	0.028	0.200 **
Stopping bullying (teachers) ^e^	−0.002	0.023	−0.003	0.002	0.025	0.003 *
Stopping bullying (students) ^f^	−0.038	0.026	−0.051	0.020	0.031	0.023
City ^g^	−0.087	0.043	−0.063 *	−0.032	0.046	−0.022
Age	0.038	0.019	0.062 *	−0.004	0.021	−0.006

* *p* < 0.05, ** *p* < 0.001, B = unstandardized regression coefficient, SE_b_ = standardized error of the coefficient, and β = standardized coefficient. ^a^ Do you consider yourself to be healthy?; ^b^ How often have you been bullied at school?; ^c^ How often have you bullied others at school?; ^d^ I feel safe at school; ^e^ Teachers or other adults at school make an effort to stop bullying; ^f^ Other students take action to stop bullying situations; ^g^ In which city do you live? Reference values: health = yes, happiness = yes, bullying = almost every day, bullying others = almost every day, safety = always, stopping bullying (teachers) = always, stopping bullying (students) = always, city = Klaipėda city and Klaipėda district, age = 17 years old.

## Data Availability

The original contributions presented in this study are included in the article. Further inquiries can be directed to the corresponding author.
